# Acceptance of 3D Printing by Occupational Therapists: An Exploratory Survey Study

**DOI:** 10.1155/2022/4241907

**Published:** 2022-12-28

**Authors:** Karin Slegers, Anna M. Krieg, Monique A. S. Lexis

**Affiliations:** ^1^Research Centre for Assistive Technology in Care, Zuyd University of Applied Sciences, Heerlen, Netherlands; ^2^Tilburg School of Humanities and Digital Sciences, Department of Communication and Cognition, Tilburg University, Tilburg, Netherlands

## Abstract

Do-it-yourself (DiY) assistive technology gained attention in accessibility literature recently, especially in relation to the rise of digital fabrication technologies, such as 3D printing. Previously, small-scale studies showed that care professionals generally respond positively towards the idea of creating DiY assistive devices for their clients. However, several barriers and concerns may hinder care professionals' actual adoption of digital fabrication technologies. To better understand occupational therapists' willingness to adopt 3D printing, we have conducted an exploratory survey study (*N* = 119) based on the unified theory of acceptance and use of technology (UTAUT). Confirming previous studies, occupational therapists in this study showed generally positive attitudes towards adopting 3D printing technology. Factors that may affect their intentions to use 3D printing technology include expectations regarding job performance, effort, social influence, and facilitating conditions, as well as one's tendency to adopt novel technologies. Furthermore, occupational therapists will likely experience difficulties when first using 3D printing technology, despite their overall positive expectations of the ease of use. Therefore, we recommend that further research should focus on training, but especially on effective ways to support occupational therapists on the job, for instance, by facilitating collaborations with 3D printing experts.

## 1. Introduction

The concept of do-it-yourself (DiY) assistive technology has gained interest in recent years, partly in response to the rise of digital fabrication technologies that hold much promise for developing tailor-made assistive devices without the involvement of commercial large-scale manufacturers. Such assistive devices may include a wide range of tools for activities of daily living, such as pencil and cutlery grips, glass or cup holders, extensions for toys or game consoles, prostheses, and orthoses. Several benefits of such DiY assistive devices have been discussed in the literature [1–12], including a better fit between products and clients' needs and wishes, customization opportunities, lower production costs, and improved access to assistive technology.

It has been suggested that care professionals have an important role in the design and production of DiY assistive devices [6, 11–13]. Occupational therapists (OTs) are particularly mentioned in this respect, as they are typically involved in providing assistive devices and therefore have essential knowledge about their clients' abilities, needs, and wishes. However, the involvement of OTs, or care professionals in general, in creating DiY assistive devices seems to remain quite low [2]. Therefore, it is important to better understand care professionals' attitudes and willingness to adopt DiY technologies to create assistive devices for their clients.

Quite a few studies have looked specifically into the potential value that 3D printing has to offer in this respect. These studies seem to consistently show positive reactions among care professionals regarding the potential benefit of DiY technologies (e.g., [2, 6, 9–12]. On the other hand, the same studies have also identified several potential barriers for the adoption of such technologies, which include lack of time [1, 3, 5, 7, 10], lack of awareness of the opportunities of digital fabrication technologies [14], lack of confidence in one's own abilities to design and print DiY assistive devices [1, 3, 7, 10, 11, 13, 14], low ease of use of 3D printing technology [1, 3, 10, 11], and concerns about the quality of DiY assistive devices [3, 6, 7].

The majority of the studies referred to were qualitative studies, or case studies with small sample sizes (typically between 4 and 13 participants), mostly situated in the United States. This study is aimed at assessing the general attitude of OTs towards 3D printing in a more quantitative way and with a larger sample size. To do this, we set out to measure OTs' behavioural intention to use 3D printing technology in their work by means of a survey based on the unified theory of acceptance and use of technology (UTAUT) by Venkatesh et al. [15]. According to this theory, the behavioural intention to use novel technology has four main determinants: users' expectations regarding how the technology will help them in their job performance (“performance expectancy” (PE)), how easy users expect it will be to adopt and use the technology (“effort expectancy” (EE)), the degree to which users believe that others think they should use the technology (“social influence” (SI)), and the extent to which users feel that organizational and technical infrastructures are in place to support them when using the technology (“facilitating conditions” (FC)). The purpose of this study was to establish to what extent these determinants, as well as several demographic factors and other expectations, play a role in OTs' intentions to use 3D printing technology. A better understanding of such factors may facilitate the development and organization of interventions to facilitate care professionals' involvement in designing and developing DiY assistive devices, e.g., by raising knowledge or awareness, by offering training regarding (the software used for) the design and production of assistive devices, and by setting up support systems and collaborations.

## 2. Materials and Methods

A cross-sectional survey study, involving both quantitative and qualitative data collection, was carried out online between May and July 2020 and was approved by the Research Ethics and Data Management Committee of Tilburg University in the Netherlands.

### 2.1. Participants

Participants for this survey study were professional OTs as well as OT students. A combination of nonprobability sampling and snowball sampling was used to approach a broad, international sample of OTs. Besides being a (future) professional OT, there were no inclusion criteria. Participants were recruited via announcements in newsletters and social media of professional associations for OTs in the Netherlands and in the region of Flanders in Belgium. Furthermore, Zuyd University of Applied Sciences in the Netherlands, which offers a study program for occupational therapy, invited OTs from their own network, including alumni currently working as OTs in Germany. Recruitment posts were also shared via several occupational therapy communities on social media such as Facebook, LinkedIn, and Thingiverse (which is a platform for sharing digital files that can be used for creating objects by means of digital manufacturing technologies). Finally, all participants were kindly asked to bring our study to the attention of colleagues who might also be interested to participate.

In total, data were collected from 127 OTs. Data from participants who had only provided demographic information via the survey was removed, which resulted in a dataset of 119 participants. Given the exploratory nature of this study, participants whose data were otherwise incomplete were not removed from the dataset, which results in differences in participant numbers for the survey items that we discuss in this paper.

### 2.2. Material and Measures

The 95-item survey (see Supplemental Material section) was conducted via the online survey tool Qualtrics. In this section, we describe the items of the survey that we have included in the analyses reported in this paper. The survey had three main sections: (1) background information, (2) acceptance of 3D printing technology and early adopter status, and (3) attitude towards 3D printing technology. The first section of the survey, addressing participants' background information, contained 3 demographic questions (about participants' age, gender, and country of residence), 4 questions about participants' experience as OTs (years of experience, work setting, area of expertise, and main client groups), and finally 16 question about participants' experience with 3D printing (of which we included the following items in this paper: years of experience, number and kind of printed objects, self-reported levels of experience, skills, difficulty and enjoyment, help needed with (specific aspects of) 3D printing, and issues encountered while learning to print 3D objects).

The second survey section started with a short introduction about how 3D printing works and what kind of objects one could create with a 3D printer (including several examples relevant to occupational therapy, such as hand grips, a prosthesis, and a wrist cast). Survey items in this second section addressed participants' acceptance of 3D printing technology as well as their early adopter status. To assess participants' acceptance of 3D printing, three statements were included about behavioural intention to use 3D printing technology (BIU). Next, several items addressed the four main determinants of this intention to use according to the UTAUT [15]: performance expectancy (PE - 8 items), effort expectancy (EE - 8 items), social influence (SI - 4 items), and facilitating conditions (FC - 7 items). These statements were all based on a previous study, in which a selection of the original UTAUT items formulated by Venkatesh et al. [15] was adapted for measuring OT's expectancies regarding 3D printing technology [12]. Besides explicitly mentioning 3D printing technology, these adaptations involved the use of future conditional tense rather than present tense, as it was anticipated that several participants would not have 3D printing facilities at their disposal yet. Furthermore, the selection of items included by Slegers et al. differed slightly from the final selection of UTAUT items that were validated by Venkatesh et al. For instance, Slegers et al. decided to leave out items referring to participants' employers or the management of the organizations they work for, because many occupational therapists are self-employed. All items that were used in this section of the survey were measured on a 5-point Likert scale.

Finally, the second survey section contained a set of 14 statements to measure participants' early adopter status (i.e., the opinion leadership and consumer novelty seeking items of the instrument developed by Chau and Hui [16]). This instrument originally used a 7-point scale, but we opted for a 5-point scale to allow for consistency in relation to the UTAUT items. We included this instrument, as we believed that one's tendency to adopt new technology in general might affect OTs' acceptance of 3D printing.

The third and final section of the survey included a collection of five 7-point semantic differential rating scales to assess participants' general attitude towards using 3D printing technology for their job, based on Davis [17]. Here, we replaced the term “electronic mail” (which was the original focus of Davis' work) with “3D printing technology.”

### 2.3. Procedure

A link to the online survey was provided in the calls for participation that were shared via newsletters and social media posts. Depending on the source of the post, the link led to a Dutch, German, or English version of the survey. Upon clicking the link, participants were first presented with information about the study's purpose, procedures, and data management and were asked to provide informed consent for using their data. Next, participants were led through all sections of the survey outlined above. After completing the survey, participants were thanked for their participation. Data were collected anonymously. Participants could submit their email addresses in case they were interested in participating in follow-up research activities, but these email addresses were removed from the dataset before analysis.

### 2.4. Analysis

Due to the small sample size and the exploratory goal of our study, analyses were mainly descriptive. We aimed to describe our sample and dataset with respect to OTs' attitudes, expectations, and intentions regarding the use of 3D printing for their job. In addition to descriptive analyses, we conducted correlational analyses to explore the relationships between several of the variables included in our study. More specifically, we were interested in exploring potential determinants of OTs' acceptance of and attitude towards 3D printing technology. Therefore, we analysed correlations between the demographic variables, the variables assessing participants' experience with 3D printing, and the variables concerning participants' acceptance of and attitudes towards 3D printing technology. We used bivariate (Pearson) correlation analysis, for which Bujang and Baharum [18] suggest a minimal sample size of 84 to achieve a considerable sizeable correlation coefficient of 0.3 with a power of 80%.

To describe our sample, the following demographic variables were extracted from the dataset: chronological age, gender, and country/continent of residence. Besides chronological age, we were interested in the concept of technology generations coined by Docampo Rama et al. [19] in order to explain age-related differences in difficulties with user interfaces. These authors showed that generation-related previous experience with certain types of technology explains variation in the number of errors people make while using interfaces, in addition to the variation explained by chronological age. Docampo Rama et al. discerned generations who grew up using mechanical interfaces (born before 1960) from generations growing up with software style interfaces (born from 1960 onwards). For 3D printing, we believed that encountering virtual and 3D environments at an early age might facilitate the creation of 3D models. As such, we classified participants in three technology generations: the electromechanical generation, the software generation, and the virtual 3D generation. The latter generation consists of participants aged 18 to 32 at the time of our study (cf. [20]).

Specific to the profession of occupational therapy, we established participants' professional status (student vs. professional), years of experience working as a professional OT, the specific setting participants work in, and their areas of expertise.

As we believed that experience with 3D printing involves both participants' actual experience and their perceived skill level, we used the average score of both the self-reported experience level and the self-reported skill level regarding 3D printing technology as an indication of experience with 3D printing in the correlation analyses. This average score showed a relatively high internal consistency (*α* = .817). In addition, we descriptively analysed several aspects of participants' experience with 3D printing: years of experience, number and types of objects printed, self-reported difficulty, and enjoyment and need for help with 3D printing.

Several other variables were calculated for use in the correlation analyses. For each main UTAUT construct (performance expectancy, effort expectancy, social influence, facilitating factors, and behavioural intention to use), we recoded the survey items and used the average scores of all construct items. All constructs showed acceptable to high internal consistency, ranging from *α* = .673 for social influence to *α* = .921 for performance expectancy. The semantic differential scales measuring general attitude towards using 3D printing in one's job showed high internal consistency (*α* = .948). For these scales, too, the average score of all items was used for further analysis. The same was done for the items measuring participants' tendency to adopt new technology, which also showed high consistency (*α* = .931).

## 3. Results

### 3.1. General Demographics

Table 1 shows information about participants' age, gender, cultural background, and tendency to adopt technology. Although all technology generations were represented in our sample, the group of participants belonging to the oldest, electromechanical generation was very small (*n* = 6). Because of this, whenever we present comparisons between generations in this section, these will only involve the software generation and the virtual 3D generation.

The majority of our participants (*n* = 81 or 68.1%) lived in Europe (especially in the Netherlands and Germany). In addition, a substantial part of our sample (*n* = 38 or 31.9%) was based in other continents. Moreover, 19 (16%) of our participants worked in countries that are classified as developing economies by the United Nations [21]. As the potential of 3D printing has been recognized as especially promising for developing economies [22], we deemed it useful to explore differences between OTs from both developing and developed economies. These differences are reported later in this section.

The average score on the early adopter status was moderate (see Table 1). No differences were found in this respect between OTs from the software generation and the virtual 3D generation nor between participants from developing and developed economies.

### 3.2. Occupational Therapy Demographics

Information about participants' occupational backgrounds is summarized in Table 2. Our sample consisted of a diverse group of mostly professional OTs working a variety of settings. Most worked in their own practice, in a collective practice, or in a rehabilitation centre. Our sample also represents a broad variety of areas of expertise, with most participants specialising in neurology, paediatrics, or geriatrics (see Table 2).

### 3.3. Experience with 3D Printing

Table 3 shows details about the experience with 3D printing of participants in our sample. About a third of the participants (31.1%) reported that they had once worked with 3D printing technology. Somewhat surprisingly, this proportion was significantly higher in the software generation (48.1%) than in the virtual 3D generation (13.1%) (*χ*^2^ (1, *N* = 113) = 16.60, *p* < .001). The same was found for the proportion of participants who had considered working with 3D printing technology: 55.6% in the software generation had considered this vs. 32.1% in the virtual 3D generation (*χ*^2^ (1, *N* = 80) = 4.11, *p* = .043). No differences were found in this respect between OTs from developing and developed economies.

Looking at the participants who reported to have some experience with 3D printing, the number of objects they had printed ranged considerably from 1 to 500. However, most of these participants had printed one or just a few objects (see Table 3).

The participants with 3D printing experience judged their own levels of experience and skills for 3D printing as rather moderate. Furthermore, although they considered 3D printing to be moderately difficult, they also reported high levels of enjoyment (see Table 3). No differences between OTs from both technology generations were found in respect to these self-reported variables. The number of participants from developing economies who had provided data in relation to these variables was too small to analyse differences between developing and developed economies.

The majority of the participants who reported some experience with 3D printing indicated that they do not feel capable of creating a 3D printed object on their own and need help (see Table 3). Twenty-six of them provided further details about the issues they encountered. They mostly reported difficulties with creating a digital 3D model (*n* = 12), lack of knowledge about material characteristics (e.g., strength, flexibility, and safety) (*n* = 9), and lack of experience in general, or with printer/software settings in particular (*n* = 8). Other issues included difficulties with objects' finish or costs. Twenty participants further explained with which aspects of the 3D printing process they needed help. Nine of them needed help with the entire process, seven only needed help to create the digital 3D model, and four needed help only with adjusting printer settings and operating the printer.

Twenty-one participants provided information about the kind of objects they had printed for their clients. The most frequently mentioned categories of objects were grips for holding/utilizing tools or other objects such as cutlery, pens, keys, and zippers (*n* = 10), adjustments to existing objects like chairs or toys (e.g., game consoles) (*n* = 6), mounts or holders (e.g., for cups or eye trackers) (*n* = 5), splints (*n* = 4), and seating or posture support (*n* = 3). Other types of objects mentioned were prostheses, therapy or exercise aids, and reading aids.

Of those participants who had never used 3D printing technology (*n* = 82), the majority (59.8%) had never considered working with 3D printing at all. When the participants who had never used 3D printing were asked about their particular reasons for not working with this technology, 34 participants (41.5%) shared information. About half of them (*n* = 24) reported that they did not have access to a printer or that they did not have the opportunity to experiment with 3D printing yet. Furthermore, several reported a lack of experience and/or knowledge (*n* = 9) or mentioned that 3D printing is too expensive (*n* = 6). Other reasons included lack of support, lack of time, and concerns about production time and productivity.

### 3.4. UTAUT and General Attitude towards 3D Printing

The mean scores for the UTAUT constructs and participants' general attitude towards using 3D printing in their job are listed in Table 4, which shows quite high levels for behavioural intention to use 3D printing and for general attitude. In addition, scores for performance expectancy, effort expectancy, and facilitating conditions are moderately high, while the average score for social influence is neutral.

### 3.5. Correlations

The correlations between demographic variables, experience with 3D printing, the UTAUT constructs, and general attitude towards 3D printing are also listed in Table 4. The correlations are mostly in line with our expectations: all four key determinants correlated positively and significantly with behavioural intention to use 3D printing technology. In addition, we found a high positive correlation between behavioural intention to use 3D printing and participants' general attitude towards using 3D printing in their job. General attitude, in turn, correlated positively with three of the key UTAUT determinants: performance expectancy, social influence, and facilitating conditions.

Behavioural intention and general attitude showed similar correlations with three of the demographic variables: gender, country classification, and early adopter status. Female participants, participants working in developing economies, and participants with higher scores on the early adopter items showed higher behavioural intentions and general attitudes. Zooming in on the individual UTAUT determinants, no significant correlations were found for gender, while participants working in developing economies as well as participants with higher early adopter statuses showed higher scores for performance expectancy, social influence, and facilitating conditions. In addition, a positive correlation was found between age and social influence, and OTs in the 3D virtual generation showed higher performance expectancy scores.

No significant correlations were found between any of the variables related to participants' experience with 3D printing on the one hand and behavioural intention, general attitude, or the key UTAUT constructs on the other hand.

## 4. Discussion and Conclusion

The exploratory study reported in this paper is aimed at further substantiating the positive reactions of OTs regarding the potential value of 3D printing technology in their job, as reported by previous small-scale studies. In addition, we aimed to understand more in-depth factors which may contribute to OTs' willingness to use 3D printing.

The findings of our study do seem to confirm the positive reactions to the potential value of 3D printing among OTs. Participants in our study showed positive general attitudes towards the idea of using 3D printing technology in occupational therapy. In addition, they seem keen to start using 3D printing technology in their own jobs. We do have to keep in mind though that our sample has likely suffered from inclusion bias. As we specifically advertised our research as a survey study about 3D printing in occupational therapy, OTs with a keen interest in 3D printing may have been more inclined to participate than OTs without such interest. Indeed, more than half of our sample reported to have experimented with 3D printing before, or to have considered this. As such, any generalizations of the positive reactions to 3D printing towards OTs in general should be done with care.

Besides the positive attitudes and high intentions to use 3D printing, the OTs in our sample reported several challenges and barriers. Participants who had some experience with 3D printing reported that they do not find 3D printing to be an easy task. Moreover, more than half of them need help when designing and printing an object. They especially have difficulties with creating digital 3D models and experience a lack of material knowledge. To successfully implement 3D printing in occupational therapy, we therefore believe it will be essential to provide fundamental support. Such support may include developing more accessible software for making digital 3D models and for operating 3D printers. In addition, dedicated training and education in conceptualizing and realizing 3D printed assistive devices might be helpful. Most importantly, we believe that setting up support systems and collaborations with 3D printing experts is a promising way forward.

A closer look at our participants' intentions and attitudes showed that the key determinants of technology acceptance according to UTAUT seem to play a role for 3D printing technology as well. OTs who believe that 3D printing would benefit them and enhance their work (cf. performance expectancy), who expect that 3D printing technology will be easy to use (cf. effort expectancy) and that their social environment thinks they should use 3D printing (cf. social influence), and who expect that sufficient support and resources would be available for them (cf. facilitating conditions) show higher intentions to use 3D printing in their job. In general, OTs in our sample show relatively high scores for each of these determining factors, except for the influence of their social environment. This may indicate that 3D printing is not yet commonly seen as an essential new technological development OTs should invest in.

The positive expectations regarding the ease of use of 3D printing technology are rather interesting, as it seems to contradict findings of several previous studies that point to steep learning curves and low self-confidence in one's own technology skills (e.g., [1, 7, 10]). In one of our own previous studies [12], OTs who had never used 3D printing before also reported positive expectations regarding the ease of use of 3D modelling and printing software. However, initial hands-on experience with such software seemed to diminish their perception of the ease of use, even to the extent that participants seriously doubted if they would ever be capable of using 3D printing technology. They also became less inclined to even try to learn. The findings of our current study, however, seem to show that even though a substantial proportion of the OTs in our sample had experience with 3D printing and reported difficulties with using this technology, this did not seem to relate to a lower intention to use 3D printing in the future. This is promising, as it may suggest that disappointing experiences with ease of use do not fully hamper OTs' positive attitudes and intentions.

The traditional demographic factors that UTAUT theorizes to affect intention to use seemed less important for OTs' intention to use 3D printing. We did find that women show higher intentions, but the fact that the large majority of OTs in the general population is female [23] makes this finding less relevant. No effects of age (or technology generation) nor of experience with 3D printing were found in our study.

Our analyses also revealed some more novel insights into factors that may play a role in OTs' intention to use 3D printing in their job. Most importantly, we found that OTs' early adopter status positively correlates with their intention to use 3D printing. As such, when aiming to enthuse OTs to engage in 3D printing, it may be worthwhile to pay special attention to those OTs who do not consider themselves to be early adopters. Somewhat surprisingly, OTs with lower early adopter statuses do not seem to have different expectations towards the ease of use of 3D printing technology. However, they seem less convinced of the benefits of 3D printing and of the support they would receive. Also, they feel less pressure from their social environment to engage in 3D printing. As such, when designing interventions to raise awareness and knowledge of digital fabrication technologies among care professionals, it might be worthwhile to explicitly address OTs' perceptions of the impact that 3D printing might have on their job and to emphasize the support and resources that will be arranged, especially when OTs with low early adopter statuses are involved.

Another interesting new insight is that OTs in developing countries show higher intentions to use 3D printing technology. We expect that this is mainly related to the high potential of 3D printing in developing economies, in which OTs' clients have less access to commercially available assistive devices compared to OTs' clients in developed economies. This is further supported by the fact that we found higher expectations towards the benefits of 3D printing for job performance for OTs in developing countries in our sample.

Some smaller findings that we find worthwhile to discuss include the fact that OTs from the 3D virtual technology generation show higher performance expectancy of 3D printing technology. This may suggest that the technologies that were available when we grew up affect our perceptions of the potential benefit of 3D printing technology. However, we find it difficult to explain this finding. It could be argued that growing up with 3D graphics in software makes people less reluctant to create 3D models themselves, but this should have translated to a relationship between technology generation and effort expectancy rather than performance expectancy.

A final finding interesting to discuss is that, in general, older OTs seem to be less convinced that their social environment believes they should use 3D printing than younger OTs. Here, too, finding an explanation is not straightforward. Perhaps, older OTs are less susceptible for peer pressure than younger OTs or older OTs tend to work in environments that exert less pressure to adopt new technologies. We can only speculate about this.

## 5. Limitations

A few limitations of this study are important to notice here. Most importantly, this concerns several issues related to the representativeness of our sample. Compared to the general population of OTs, our sample included a relatively large proportion of male participants. In the Netherlands, for instance, 6.3% of OTs is male (based on statistics from 2014 [23]), while 16.0% of our sample (19 participants) were identified as male. As the proportion of male participants is still rather small, we do not believe that this has caused a serious representation bias.

A more serious limitation may be caused by the fact that our dataset mostly included data from OTs in Europe (or even more specific: in The Netherlands and Germany—in line with the countries of origin of the authors of this paper). As such, a cultural bias may have occurred: we cannot rule out that our findings are somewhat specific to the Dutch and German context.

A more important bias, however, is related to the way we promoted our study, as our calls for participation specifically mentioned that the survey addressed 3D printing in occupational therapy. Therefore, it is likely that our study suffered from an inclusion bias, with an overrepresentation of OTs with higher-than-average interest in 3D printing.

Another limitation of our study concerns sample size. Our sample was too small to analyse predicting relationships between variables, so we were only able to assess and interpret correlational relationships. Therefore, our study should be considered as an exploratory study, and the findings should be interpreted with care.

Finally, this study is subject to some methodological limitations. The fact that we adapted the scales of the early adopter status, statements in our survey may have caused a methodological limitation. Although we have no reason to suspect that changing the 7-point scale that was used by Chau and Hui [16] to a 5-point scale did indeed introduce methodological issues, and we have not assessed the validity of our adapted items. Another potential concern is the fact that some participants may have filled out the survey more than once, although a manual check of highly similar response patterns did not reveal any suspicious entries.

## 6. Conclusion

In conclusion, this study provides additional support for the positive reactions among OTs to the idea of adopting 3D printing technology for creating assistive devices that was reported in smaller studies in the past few years. It shows that this positivism is not limited to OTs located in the United States. We consider this to be good news, as the potential benefit of 3D printed assistive devices has been increasingly recognized in academic literature. The fact that OTs in practice have similar positive expectations, attitudes and intentions are promising for the uptake of 3D printing technology in health care.

In addition, our study contributes new insights about the factors that seem to affect OTs' intentions to use 3D printing. This is a valuable knowledge especially in terms of initiatives that are employed to stimulate OTs to engage in 3D printing, such as awareness or knowledge campaigns, training, support systems, and collaborations. In addition, even though OTs seem to have positive expectations towards the ease of use of 3D printing technology, our (and previous) findings also show that they are likely to encounter difficulties with using the technology. Therefore, we recommend that further research should focus on training, especially on effective ways to support OTs on the job. As several other authors did [4, 7, 11, 14], we previously suggested [12] that collaborations between OTs and 3D printing experts might be a suitable way forward, and we believe the findings of this study support this suggestion.

## Figures and Tables

**Table 1:** Demographic information about participants.

Age	
Chronological age (in years)	
Range	19–75
Mean (SD)	35 (12.69)
Technology generations (*n*, %)	
Electromechanical generation	6 (5.0)
Software generation	52 (44.7)
Virtual 3D generation	61 (51.3)

Gender (*n*, %)	
Male	19 (16.0%)
Female	98 (82.4%)
Other	1 (0.8%)
Undisclosed	1 (0.8%)

Background (*n*, %)	
Continent of residence	
Europe	81 (68.1%)
North America	5 (4.2%)
Australia and New Zealand	14 (11.8%)
Asia	4 (3.3%)
South America	1 (0.8%)
Africa	14 (11.8%)
Developing economies	19 (16%)
Developed economies	100 (84%)

Early adopter status (mean, SD)	
Tendency to adopt new technology (range 1-5)	2.80 (.85)

**Table 2:** Occupational background of the participants.

Experience	
Status (*n*, %)	
Student	18 (15.1%)
Professional OT	101 (84.9%)
Working experience of professional participants (in years)	
Range	0–53
Mean (SD)	13.33 (11.36)
Work context	
Setting^∗^ (*n*, %)	
Rehabilitation centre	33 (27.7%)
Nursing home or care home	13 (10.9%)
Hospital	13 (10.9%)
Care facility for people with disabilities	7 (5.9%)
Mental health care facility	6 (5.0%)
Own practice	17 (14.3%)
Collective practice for primary care	15 (12.6%)
School (regular or special education)	12 (10.1%)
Day activity centre	5 (4.2%)
Other extramural organizations (incl. governments)	13 (10.9%)
OT teacher	15 (12.6%)
Other	5 (4.2%)
Area of expertise^∗^	
Neurology	50 (42.0%)
Paediatrics	46 (38.7%)
Geriatrics	33 (27.7%)
Orthopaedics	28 (23.5%)
Hand injuries	24 (20.2%)
Complex/multiple intellectual disabilities	22 (18.5%)
Other (psychiatric/mental health care, chronic pain, rheumatology, oncology, cardiology, surgery, and lung diseases)	77 (64.7%)

**Table 3:** Details on experience with 3D printing of the participants.

Proportions of participants with 3D printing experience (*n*, %)	
Once worked with 3D printing technology	37 (31.1%)
Never worked with 3D printing technology	82 (68.9%)
Considered working with 3D printing technology	33 (40.2%)
Never considered working with 3D printing technology	49 (59.8%)

Actual experience with 3D printing (*n* = 37)	
Years of experience (*n*, %)	
1 year or less	18 (49.6%)
2 years	6 (16.2%)
3 years	3 (8.1%)
4 years	4 (10.8%)
Longer than 4 years	6 (16.2%)
Number of objects printed (information provided by *n* = 31) (*n*, %)	
1	6 (19.4%)
2-5	14 (45.2%)
>5	11 (35.5%)

Self-reported experience with 3D printing (*n* = 37)	
Self-reported levels of (mean, SD)	
Experience (range 1-5)	2.54 (1.17)
Skill level (range 1-5)	2.54 (1.07)
Enjoyment (range 1-5)	4.05 (.70)
Difficulty (range 1-5)	3.14 (SD.95)
Feeling capable of creating 3D printed objects on their own (*n*, %)	8 (21.6%)
Need help with creating 3D printed objects (*n*, %)	27 (73.0%)

**Table 4:** Correlations between demographic variables, experience with 3D printing, UTAUT constructs, and general attitude.

Variables	*n ^b^*	*M*	SD	1	2	3	4	5	6	7	8	9	10	11	12	13	14	15
Demographic variables																		
(1) Age	119	35.64	12.69	—	—	—	—	—	—	—	—	—	—	—	—	—	—	—
(2) Gender (female vs. male)	117	n/a	n/a	-.04	—	—	—	—	—	—	—	—	—	—	—	—	—	—
(3) Country classification (developed vs. developing economy)	119	n/a	n/a	-.21^∗^	-.37^∗∗^	—	—	—	—	—	—	—	—	—	—	—	—	—
(4) Technology generation (3D virtual vs. software)	113	n/a	n/a	.83^∗∗^	-.06	-.13	—	—	—	—	—	—	—	—	—	—	—	—
(5) Early adopter status (1-5)	103	2.80	0.85	-.21	-.26^∗∗^	.37^∗∗^	.01	—	—	—	—	—	—	—	—	—	—	—

Experience with 3D printing																		
(6) Years of experience with 3D printing	101	13.33	11.36	.94^∗∗^	.08	-.33^∗∗^	.79^∗^	.04	—	—	—	—	—	—	—	—	—	—
(7) Number of objects printed	31	2.29	0.94	-.41^∗^	-.05	.02	-.09	-.14	-.45^∗^	—	—	—	—	—	—	—	—	—
(8) Self-reported level of experience with 3D printing (1-5)	37	2.54	1.17	-.14	-.03	.14	.05	-.05	-.02	.47^∗∗^	—	—	—	—	—	—	—	—
(9) Self-reported skill level regarding 3D printing (1-5)	37	2.54	1.07	-.12	-.27	.32	.06	.00	-.06	.28	.69^∗∗^	—	—	—	—	—	—	—

UTAUT constructs and general attitude																		
(10) Behavioural intention to use 3D printing in one's job (BIU) (1-5)	105	3.55	0.91	.09	-.20^∗^	.42^∗∗^	.07	.50^∗∗^	.01	.23	-.06	-.08	—	—	—	—	—	—
(11) Performance expectancy (PE) (1-5)	117	3.40	0.83	-.06	-.14	.47^∗∗^	-.19^∗^	.30^∗∗^	-.10	.08	-.01	-.06	.62^∗∗^	—	—	—	—	—
(12) Effort expectancy (EE) (1-5)	110	3.49	0.54	-.08	.00	.09	-.10	.03	-.14	.10	.00	.11	.23^∗^	.19	—	—	—	—
(13) Social influence (SI) (1-5)	110	2.76	0.73	.21^∗^	-.14	.28^∗^	.18	.48^∗∗^	.18	.00	-.04	-.09	.62^∗∗^	.60^∗∗^	.09	—	—	—
(14) Facilitating conditions (FC) (1-5)	107	3.21	0.63	.10	-.13	.23^∗^	-.06	.47^∗∗^	.07	.00	-.05	-.05	.62^∗∗^	.56^∗∗^	.15	.57^∗∗^	—	—
(15) General attitude towards using 3D printing in one's job (1-7)	100	5.63	1.11	.17	-.27^∗∗^	.26^∗^	.09	.34^∗∗^	.11	.21	.27	.26	.57^∗∗^	.61^∗∗^	.15	.54^∗∗^	.52^∗∗^	—

^a^Grey cells indicate significant correlations (^∗^*p* < .05; ^∗∗^*p* < .01). ^b^This column shows the number of participants who answered all scale items for the constructs.

## Data Availability

The survey data used to support the findings of this study have not been made available due to the exploratory nature, the nonrandom sampling, and the very specific focus of the study.

## Supplementary Materials

Supplementary Materials The table shows all items of the survey that was used in the study reported in this paper as well as the answer options. Items marked with an asterisk were included in the analyses performed for this paper.

## References

[1] Buehler E., Kane S. K., Hurst A. (2014). ABC and 3D: opportunities and obstacles to 3D printing in special education environments. *ASSETS14 - proceedings of the 16th international ACM SIGACCESS conference on computers and accessibility*.

[2] Buehler E., Branham S., Ali A. (2015). Sharing is caring: assistive technology designs on thingiverse. *conference on human factors in computing systems - proceedings*.

[3] Buehler E., Comrie N., Hofmann M., McDonald S., Hurst A. (2016). Investigating the implications of 3D printing in special education. *Computing*.

[4] de Couvreur L., Goossens R. (2011). Design for (every)one: co-creation as a bridge between universal design and rehabilitation engineering. *CoDesign*.

[5] Hofmann M., Burke J., Pearlman J. (2016). Clinical and maker perspectives on the design of assistive technology with rapid prototyping technologies. *proceedings of the 18th international ACM SIGACCESS conference on computers and accessibility - ASSETS ‘16*.

[6] Hofmann M., Williams K., Kaplan T. (2019). “Occupational therapy is making”: clinical rapid prototyping and digital fabrication. *conference on human factors in computing systems - proceedings*.

[7] Hook J., Verbaan S., Durrant A., Olivier P., Wright P. (2014). A study of the challenges related to DIY assistive technology in the context of children with disabilities. *Proceedings of the 2014 conference on designing interactive systems - DIS ‘14*.

[8] Hurst A. (2018). Making ‘making’ accessible. *Proceedings of the internet of accessible things on - W4A ‘18*.

[9] Hurst A., Tobias J. (2011). Empowering individuals with do-it-yourself assistive technology. *The proceedings of the 13th international ACM SIGACCESS conference on computers and accessibility - ASSETS ‘11*.

[10] McDonald S., Comrie N., Buehler E. (2016). Uncovering challenges and opportunities for 3D printing assistive technology with physical therapists. *Proceedings of the 18th international ACM SIGACCESS conference on computers and accessibility - ASSETS ‘16*.

[11] Moraiti A., Vanden Abeele V., Vanroye E., Geurts L. (2015). Empowering occupational therapists with a DIY-toolkit for smart soft objects. *Proceedings of the ninth international conference on tangible, embedded, and embodied interaction - TEI ‘14*.

[12] Slegers K., Kouwenberg K., Loučova T., Daniels R. (2020). Makers in healthcare: the role of occupational therapists in the design of DIY assistive technology. *Conference on human factors in computing systems - proceedings*.

[13] Alharbi R., Ng A., Alharbi R., Hester J. (2020). ‘i am not an engineer’: understanding how clinicians design & alter assistive technology. *conference on human factors in computing systems - proceedings*.

[14] Aflatoony L., Lee S. J. (2020). CODEA: a framework for co-designing assistive technologies with occupational therapists, industrial designers, and end-users with mobility impairments. *Proceedings of the DESIGN society: DESIGN conference 1. Cambridge university press (CUP): 1843–1852*.

[15] Venkatesh V., Morris M. G., Davis G. B., Davis F. D. (2003). User acceptance of information technology: toward a unified view. *MIS Quarterly*.

[16] Chau P. Y. K., Hui K. L. (1998). Identifying early adopters of new IT products: a case of Windows 95. *Information and Management*.

[17] Davis F. D. (1993). User acceptance of information technology: system characteristics, user perceptions and behavioral impacts. *International journal of man-machine studies*.

[18] Bujang M. A., Baharum N. (2016). Sample size guideline for correlation analysis. *World Journal of Social Science Research*.

[19] Docampo Rama M., Ridder H., Bouma H. (2001). Technology generation and age in using layered user interfaces. *International Society for Gerontechnology (ISG)*.

[20] Spence J. (2008). Virtual worlds research: consumer behavior in virtual worlds. *Journal of Virtual Worlds Research*.

[21] United Nations (2020). *World Economic Situation and Prospects 2020*.

[22] Ishengoma F. R., Mtaho A. B. (2014). 3D printing: developing countries perspectives. *International Journal of Computer Applications*.

[23] Van Hassel D. T. P., Kenens R. J. (2014). *Cijfers uit de registratie van ergotherapeuten: peiling 1 januari 2014*.

