# Supplementary material for: Acceptance of 3D Printing by Occupational Therapists: An Exploratory Survey Study
Source: Occup Ther Int. 2022 Dec 28;2022:4241907. doi: 10.1155/2022/4241907 (PMC9812607; doi:10.1155/2022/4241907)
Supplement: Supplementary file 1 [file 4241907.f1.docx]

*	I expect that it would be easy for me to become skillful at using 3D printing technology.	5-point Likert cale ranging from "Strongly disagree" to "Strongly agree"
	I would find 3D printing technology easy to use.	5-point Likert cale ranging from "Strongly disagree" to "Strongly agree"
*	Using 3D printing technology would take too much time from my normal duties.	5-point Likert cale ranging from "Strongly disagree" to "Strongly agree"
	Working with 3D printing technology would be so complicated; it would be difficult to understand what is going on.	5-point Likert cale ranging from "Strongly disagree" to "Strongly agree"
*	Using 3D printing technology would involve too much time doing mechanical operations (e.g., data input).	5-point Likert cale ranging from "Strongly disagree" to "Strongly agree"
*	It would take too long to learn how to use 3D printing technology to make it worth the effort.	5-point Likert cale ranging from "Strongly disagree" to "Strongly agree"
	**UTAUT: Social influence (SI)**	
*	People who are important to me think that I should use 3D printing technology.	5-point Likert cale ranging from "Strongly disagree" to "Strongly agree"
*	I would use 3D printing technology because of the proportion of co-workers who use the system.	5-point Likert cale ranging from "Strongly disagree" to "Strongly agree"
	In general, I believe that the organization I work in would support the use of 3D printing technology.	5-point Likert cale ranging from "Strongly disagree" to "Strongly agree"
*	I believe that people in my field who would use 3D printing technology would have more prestige than those who would not.	5-point Likert cale ranging from "Strongly disagree" to "Strongly agree"
	Survey items	Answer options
	**UTAUT: Facilitating conditions (FC)**	
*	I believe I would have control over using 3D printing technology.	5-point Likert cale ranging from "Strongly disagree" to "Strongly agree"
*	I believe I would have the resources necessary to use 3D printing technology.	5-point Likert cale ranging from "Strongly disagree" to "Strongly agree"
*	I believe I would have the knowledge necessary to use 3D printing technology.	5-point Likert cale ranging from "Strongly disagree" to "Strongly agree"
*	A specific person (or group) would be available for assistance with 3D printing technology difficulties.	5-point Likert cale ranging from "Strongly disagree" to "Strongly agree"
*	Specialized instruction concerning 3D printing technology would be available to me.	5-point Likert cale ranging from "Strongly disagree" to "Strongly agree"
*	Using 3D printing technology is compatible with all aspects of my work.	5-point Likert cale ranging from "Strongly disagree" to "Strongly agree"
*	Using 3D printing technology fits into my work style.	5-point Likert cale ranging from "Strongly disagree" to "Strongly agree"
	**UTAUT: Self-efficacy (SE)**	
	I could complete a job or task using 3D print technology…	
	if there was no one around to tell me what to do as I go.	5-point Likert cale ranging from "Strongly disagree" to "Strongly agree"
	if I could call someone for help if I got stuck.	5-point Likert cale ranging from "Strongly disagree" to "Strongly agree"
	if I had a lot of time to complete the job for which the technology was provided.	5-point Likert cale ranging from "Strongly disagree" to "Strongly agree"
	if I had just the built-in help facility for assistance.	5-point Likert cale ranging from "Strongly disagree" to "Strongly agree"
	if I had only the software manuals for reference.	5-point Likert cale ranging from "Strongly disagree" to "Strongly agree"
	if I had seen someone else using it before trying it myself.	5-point Likert cale ranging from "Strongly disagree" to "Strongly agree"
	if someone else had helped me get started.	5-point Likert cale ranging from "Strongly disagree" to "Strongly agree"
	**UTAUT: Anxiety (ANX)**	
	I feel apprehensive about using the technology.	5-point Likert cale ranging from "Strongly disagree" to "Strongly agree"
	I would hesitate to use 3D printing technology for fear of making mistakes I could not correct.	5-point Likert cale ranging from "Strongly disagree" to "Strongly agree"
	I expect 3D printing technology to be somewhat intimidating to me.	5-point Likert cale ranging from "Strongly disagree" to "Strongly agree"
	Using 3D printing technology does not scare me at all.	5-point Likert cale ranging from "Strongly disagree" to "Strongly agree"
	Working with 3D printing technology would make me very nervous.	5-point Likert cale ranging from "Strongly disagree" to "Strongly agree"
	I get a sinking feeling when I think of trying to use 3D printing technology.	5-point Likert cale ranging from "Strongly disagree" to "Strongly agree"
	**Attitude**	
	Using 3D printing technology is a good idea.	5-point Likert cale ranging from "Strongly disagree" to "Strongly agree"
	I expect using 3D printing technology to be enjoyable.	5-point Likert cale ranging from "Strongly disagree" to "Strongly agree"
	3D printing technology would make my work more interesting.	5-point Likert cale ranging from "Strongly disagree" to "Strongly agree"
	3D printing technology is okay for some jobs, but not the kind of job I do. (reversed item)	5-point Likert cale ranging from "Strongly disagree" to "Strongly agree"
	I look forward to those aspects of my job that require me to use 3D printing technology.	5-point Likert cale ranging from "Strongly disagree" to "Strongly agree"
	I expect that using 3D printing technology will be frustrating for me. (reversed item)	5-point Likert cale ranging from "Strongly disagree" to "Strongly agree"
	**UTAUT: Behavioral Intention to use the system (BIU)**	
*	I plan to use or intensify the use of 3D printing technology in the future.	5-point Likert cale ranging from "Strongly disagree" to "Strongly agree"
*	I intend to encourage others to use 3D printing technology.	5-point Likert cale ranging from "Strongly disagree" to "Strongly agree"
*	I intend to be informed about 3D printing technology and use it in the future.	5-point Likert cale ranging from "Strongly disagree" to "Strongly agree"
	**Intention to buy**	
	I feel quite certain of the benefits I could expect to get if I adopted 3D printing technology.	5-point Likert cale ranging from "Strongly disagree" to "Strongly agree"
	I'm quite sure of what the relevant trade-offs are among the costs and benefits of using 3D printing technology.	5-point Likert cale ranging from "Strongly disagree" to "Strongly agree"
	I'll have to change my behavior significantly to attain the potential benefits of 3D printing technology.	5-point Likert cale ranging from "Strongly disagree" to "Strongly agree"
	Using 3D printing technology would allow me to do things that I can't easily do now.	5-point Likert cale ranging from "Strongly disagree" to "Strongly agree"
	**General attitude towards using 3D printing in one's job**	
*	All things considered, I think using 3D printing technology for my job would be:	
*	Good ….Bad	7-point semantical differential scale
*	Wise …. Foolish	7-point semantical differential scale
*	Favorable …. Unfavorable	7-point semantical differential scale
*	Beneficial …. Harmful	7-point semantical differential scale
*	Positive …. Negative	7-point semantical differential scale
	**Early adopter status**	
*	My opinions on hardware/software products seem not to count with other people	5-point Likert cale ranging from "Strongly disagree" to "Strongly agree"
*	When other people choose to adopt a hardware/software product, they turn to me for advice	5-point Likert cale ranging from "Strongly disagree" to "Strongly agree"
*	Other people select hardware/software products rarely based on what I have suggested to them	5-point Likert cale ranging from "Strongly disagree" to "Strongly agree"
*	I often persuade other people to adopt the hardware/software products that I like	5-point Likert cale ranging from "Strongly disagree" to "Strongly agree"

*	Using 3D printing technology would take too much time from my normal duties.	5-point Likert cale ranging from "Strongly disagree" to "Strongly agree"
	Working with 3D printing technology would be so complicated; it would be difficult to understand what is going on.	5-point Likert cale ranging from "Strongly disagree" to "Strongly agree"
*	Using 3D printing technology would involve too much time doing mechanical operations (e.g., data input).	5-point Likert cale ranging from "Strongly disagree" to "Strongly agree"
*	It would take too long to learn how to use 3D printing technology to make it worth the effort.	5-point Likert cale ranging from "Strongly disagree" to "Strongly agree"
	**UTAUT: Social influence (SI)**	
*	People who are important to me think that I should use 3D printing technology.	5-point Likert cale ranging from "Strongly disagree" to "Strongly agree"
*	I would use 3D printing technology because of the proportion of co-workers who use the system.	5-point Likert cale ranging from "Strongly disagree" to "Strongly agree"
	In general, I believe that the organization I work in would support the use of 3D printing technology.	5-point Likert cale ranging from "Strongly disagree" to "Strongly agree"
*	I believe that people in my field who would use 3D printing technology would have more prestige than those who would not.	5-point Likert cale ranging from "Strongly disagree" to "Strongly agree"
	Survey items	Answer options
	**UTAUT: Facilitating conditions (FC)**	
*	I believe I would have control over using 3D printing technology.	5-point Likert cale ranging from "Strongly disagree" to "Strongly agree"
*	I believe I would have the resources necessary to use 3D printing technology.	5-point Likert cale ranging from "Strongly disagree" to "Strongly agree"
*	I believe I would have the knowledge necessary to use 3D printing technology.	5-point Likert cale ranging from "Strongly disagree" to "Strongly agree"
*	A specific person (or group) would be available for assistance with 3D printing technology difficulties.	5-point Likert cale ranging from "Strongly disagree" to "Strongly agree"
*	Specialized instruction concerning 3D printing technology would be available to me.	5-point Likert cale ranging from "Strongly disagree" to "Strongly agree"
*	Using 3D printing technology is compatible with all aspects of my work.	5-point Likert cale ranging from "Strongly disagree" to "Strongly agree"
*	Using 3D printing technology fits into my work style.	5-point Likert cale ranging from "Strongly disagree" to "Strongly agree"
	**UTAUT: Self-efficacy (SE)**	
	I could complete a job or task using 3D print technology…	
	if there was no one around to tell me what to do as I go.	5-point Likert cale ranging from "Strongly disagree" to "Strongly agree"
	if I could call someone for help if I got stuck.	5-point Likert cale ranging from "Strongly disagree" to "Strongly agree"
	if I had a lot of time to complete the job for which the technology was provided.	5-point Likert cale ranging from "Strongly disagree" to "Strongly agree"
	if I had just the built-in help facility for assistance.	5-point Likert cale ranging from "Strongly disagree" to "Strongly agree"
	if I had only the software manuals for reference.	5-point Likert cale ranging from "Strongly disagree" to "Strongly agree"
	if I had seen someone else using it before trying it myself.	5-point Likert cale ranging from "Strongly disagree" to "Strongly agree"
	if someone else had helped me get started.	5-point Likert cale ranging from "Strongly disagree" to "Strongly agree"
	**UTAUT: Anxiety (ANX)**	
	I feel apprehensive about using the technology.	5-point Likert cale ranging from "Strongly disagree" to "Strongly agree"
	I would hesitate to use 3D printing technology for fear of making mistakes I could not correct.	5-point Likert cale ranging from "Strongly disagree" to "Strongly agree"
	I expect 3D printing technology to be somewhat intimidating to me.	5-point Likert cale ranging from "Strongly disagree" to "Strongly agree"
	Using 3D printing technology does not scare me at all.	5-point Likert cale ranging from "Strongly disagree" to "Strongly agree"
	Working with 3D printing technology would make me very nervous.	5-point Likert cale ranging from "Strongly disagree" to "Strongly agree"
	I get a sinking feeling when I think of trying to use 3D printing technology.	5-point Likert cale ranging from "Strongly disagree" to "Strongly agree"
	**Attitude**	
	Using 3D printing technology is a good idea.	5-point Likert cale ranging from "Strongly disagree" to "Strongly agree"
	I expect using 3D printing technology to be enjoyable.	5-point Likert cale ranging from "Strongly disagree" to "Strongly agree"
	3D printing technology would make my work more interesting.	5-point Likert cale ranging from "Strongly disagree" to "Strongly agree"
	3D printing technology is okay for some jobs, but not the kind of job I do. (reversed item)	5-point Likert cale ranging from "Strongly disagree" to "Strongly agree"
	I look forward to those aspects of my job that require me to use 3D printing technology.	5-point Likert cale ranging from "Strongly disagree" to "Strongly agree"
	I expect that using 3D printing technology will be frustrating for me. (reversed item)	5-point Likert cale ranging from "Strongly disagree" to "Strongly agree"
	**UTAUT: Behavioral Intention to use the system (BIU)**	
*	I plan to use or intensify the use of 3D printing technology in the future.	5-point Likert cale ranging from "Strongly disagree" to "Strongly agree"
*	I intend to encourage others to use 3D printing technology.	5-point Likert cale ranging from "Strongly disagree" to "Strongly agree"
*	I intend to be informed about 3D printing technology and use it in the future.	5-point Likert cale ranging from "Strongly disagree" to "Strongly agree"
	**Intention to buy**	
	I feel quite certain of the benefits I could expect to get if I adopted 3D printing technology.	5-point Likert cale ranging from "Strongly disagree" to "Strongly agree"
	I'm quite sure of what the relevant trade-offs are among the costs and benefits of using 3D printing technology.	5-point Likert cale ranging from "Strongly disagree" to "Strongly agree"
	I'll have to change my behavior significantly to attain the potential benefits of 3D printing technology.	5-point Likert cale ranging from "Strongly disagree" to "Strongly agree"
	Using 3D printing technology would allow me to do things that I can't easily do now.	5-point Likert cale ranging from "Strongly disagree" to "Strongly agree"
	**General attitude towards using 3D printing in one's job**	
*	All things considered, I think using 3D printing technology for my job would be:	
*	Good ….Bad	7-point semantical differential scale
*	Wise …. Foolish	7-point semantical differential scale
*	Favorable …. Unfavorable	7-point semantical differential scale
*	Beneficial …. Harmful	7-point semantical differential scale
*	Positive …. Negative	7-point semantical differential scale
	**Early adopter status**	
*	My opinions on hardware/software products seem not to count with other people	5-point Likert cale ranging from "Strongly disagree" to "Strongly agree"
*	When other people choose to adopt a hardware/software product, they turn to me for advice	5-point Likert cale ranging from "Strongly disagree" to "Strongly agree"
*	Other people select hardware/software products rarely based on what I have suggested to them	5-point Likert cale ranging from "Strongly disagree" to "Strongly agree"
*	I often persuade other people to adopt the hardware/software products that I like	5-point Likert cale ranging from "Strongly disagree" to "Strongly agree"
